# Questioning on consistency of a Stagnation Scale in Medication Overuse Headache: one more added to a plea of emperor’s clothes?

**DOI:** 10.1186/s10194-015-0498-4

**Published:** 2015-02-11

**Authors:** Marco Innamorati, Maurizio Pompili, Paolo Martelletti

**Affiliations:** Department of Neurosciences, Mental Health and Sensory Organs, Suicide Prevention Center, Sant’Andrea Hospital, Sapienza University of Rome, Rome, Italy; Department of Clinical and Molecular Medicine, Sapienza University of Rome and Regional Referral Headache Centre, Sant’Andrea Hospital, Rome, Italy

## Correspondence/Findings

With great pleasure we write this Reply letter to the considerations, all of them indeed very interesting and thought-provoking, from Crombez relatively to a paper of validation of an existing instrument suitable/appropriate to our vision of a chronic migraine patient’s complexity [1].

The author uses a much ironic and opinionated style [2], obviously supported from his undisputed experience in the study of psychology of pain but with an undisputed limited experience in the study of chronic migraine and its complications (such as Medication Overuse Headache [MOH]) as well [3,4], a clinical area very variegated with multiple medical comorbidities, not only psychopathological ones [5]. His letter is specifically premeditated, providing a pulpit to widen the debate on the questioned attempt [4].

Firstly, the validity of the original test, the Stagnation Scale, has been widely confirmed and it is not our duty to re-discuss it, but maybe only to choose what to validate, its Italian version. Let’s proceed with order.

The presence of emotional disturbances has been used to distinguish complicated cases of MOH (MOH Type II) [6-8] and although, it is necessary to screen MOH patients for anxiety and depression [9] psychological pain presentation might be multifaceted. Stagnation is a traditional Chinese medicine syndrome characterized by a cluster of mind/body obstruction-like symptoms such as feeling that something is stuck in the throat, chest and stomach, preoccupation or fear of losing what one possesses, and/or being unable to let go of some matters [10]. The construct of stagnation may capture some aspects of the psychological pain experienced from the individual that other Western construct such as depression, anxiety or somatization are not able to capture.

Ng et al. [11-14] operationalized the construct of stagnation through the development of the Stagnation Scale with the help of experts in traditional Chinese medicine [11]. In their initial studies, the authors obtained data suggesting that Stagnation is a clinical syndrome distinct from depression [11]. Following the studies conducted on Chinese samples, we hypothesized the utility of the Stagnation Scale also in patients with chronic headache and conducted a pilot study in a small sample of chronic migraine patients [15]. This study indicated that stagnation severity was associated with higher perceived disability independent of the severity of depression, and that it could be useful for predicting perceived disability among patients with chronic migraine [15]. These promising results suggested us to study the psychometric properties of the Stagnation Scale, the first step in the utilization of this scale to study the usefulness of the construct of Stagnation in larger samples of chronic headache patients.

Lastly, without using journalistic exhumation of false and non-existing emperor’s new clothes, we do not believe we are able to scare anyone to the point of being silent in front of a blatant fake. Therefore we thank the author for giving us the possibility to do justice to the concept that we shall not loose the spirit, curiosity and courage to look far, considering chronic migraine not like an assemblage of sealed compartments but as a perfect model for a multimodal approach.

In conclusion, it is more than certain that doing more does not mean doing better, but it is also true that as Marcel Proust said “even the wisest of doctors are relying on scientific truths the errors of which will be recognized within a few years”. We accept these limits, totally.
